# Preserving Yeast Genetic Heritage through DNA Damage Checkpoint Regulation and Telomere Maintenance

**DOI:** 10.3390/biom2040505

**Published:** 2012-10-29

**Authors:** Veronica Baldo, Jason Liang, Guoliang Wang, Huilin Zhou

**Affiliations:** 1 Ludwig Institute for Cancer Research, University of California at San Diego, La Jolla, CA 92093, USA; Email: vbaldo@ucsd.edu (V.B.); jliang@ucsd.edu (J.L.); guw001@ucsd.edu (G.W.); 2 Department of Chemistry and Biochemistry, University of California at San Diego, La Jolla, CA 92093, USA

**Keywords:** genome maintenance, DNA damage checkpoint, DNA damage response, double strand breaks (DSB), telomere

## Abstract

In order to preserve genome integrity, extrinsic or intrinsic DNA damages must be repaired before they accumulate in cells and trigger other mutations and genome rearrangements. Eukaryotic cells are able to respond to different genotoxic stresses as well as to single DNA double strand breaks (DSBs), suggesting highly sensitive and robust mechanisms to detect lesions that trigger a signal transduction cascade which, in turn, controls the DNA damage response (DDR). Furthermore, cells must be able to distinguish natural chromosomal ends from DNA DSBs in order to prevent inappropriate checkpoint activation, DDR and chromosomal rearrangements. Since the original discovery of *RAD9*, the first DNA damage checkpoint gene identified in *Saccharomyces cerevisiae*, many genes that have a role in this pathway have been identified, including *MRC1*, *MEC3*, *RAD24*, *RAD53*, *DUN1*, *MEC1* and *TEL1*. Extensive studies have established most of the genetic basis of the DNA damage checkpoint and uncovered its different functions in cell cycle regulation, DNA replication and repair, and telomere maintenance. However, major questions concerning the regulation and functions of the DNA damage checkpoint remain to be answered. First, how is the checkpoint activity coupled to DNA replication and repair? Second, how do cells distinguish natural chromosome ends from deleterious DNA DSBs? In this review we will examine primarily studies performed using *Saccharomyces cerevisiae* as a model system.

## 1. Introduction: The Importance of Genome Stability

The genetic heritage of every single cell has to be faithfully transmitted across generations in order to allow cell survival, normal cell growth and the survival of species. The loss of genomic integrity could cause chromosomal aberrations and cancer [1,2,3,4,5,6,7,8] and stable genome rearrangements have been demonstrated as inherited mutations that cause a number of other human diseases [7,9,10]. The genomic integrity of cells is constantly being endangered by DNA insults arising from endogenous stresses resulting from DNA replication errors and byproducts of cellular metabolism, such as reactive oxygen species, as well as from exogenous sources, including ionizing and ultraviolet radiation and genotoxic agents in general [11]. The ability to effectively deal with spontaneous- or environmentally-induced DNA damage is crucial for cellular survival and the maintenance of genomic stability.

**Figure 1:**
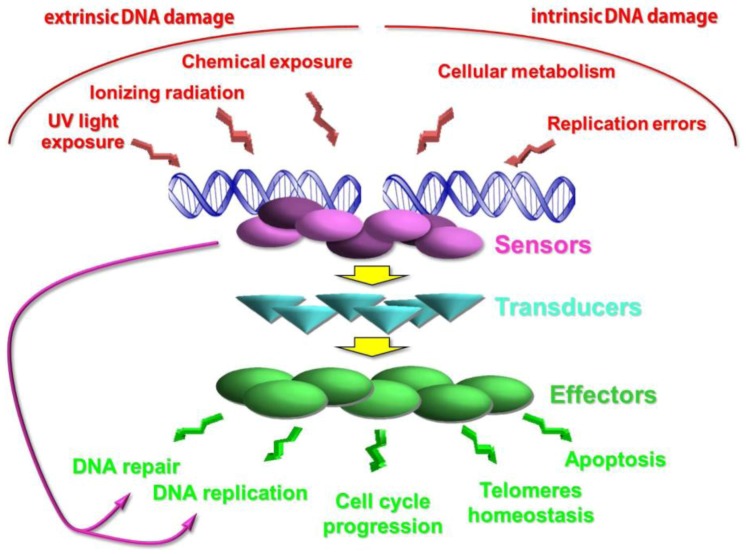
Schematic representation of the DNA damage response.

In order to preserve genome integrity cells have evolved a sophisticated surveillance mechanism called DNA damage checkpoint [12,13] that monitors the successful completion of DNA replication and initiate a coordinated cellular response when DNA damage occurs [2,14,15,16,17,18]. The DNA damage checkpoint is a signal transduction cascade that is activated in response to DNA damage and through sensors, transducers and effectors it is able to control cell cycle progression, DNA replication, transcription and repair and cellular senescence or programmed cell death in higher eukaryotes [19] (Figure 1).

The first evidence that cell cycle arrest caused by DNA damage is due to a genetically controlled mechanism and not to the damage itself came from studies carried out in *Escherichia coli*, where it was found that mutations in certain genes relieved the septation block caused by DNA damage [20]. Later, Weinert and Hartwell found that *RAD9* gene is able to control the cell cycle in response to DNA damage in *Saccharomyces cerevisiae.* This discovery led to a number of studies on the identification and characterization of many checkpoint genes in this eukaryotic microorganism, genetically well-known and easy to handle [12,17].

Here we are going to summarize studies on DNA damage checkpoint performed using *S. cerevisiae* as a model system.

## 2. The DNA Damage Checkpoint in *S. cerevisiae*

The DNA damage checkpoint has been highly conserved during evolution. In fact, most of the key players in the checkpoint response in *S. cerevisiae* identified in the last 30 years have structural and functional counterparts in *Schizosaccharomyces pombe* and humans (Table 1). The similarity among these pathways from yeast to mammal and, in particular, human cells enables their studies in a simpler organism like *S. cerevisiae*. This helps to understand the DNA damage checkpoint in more complex organisms like humans and how its deregulation leads to cancer. Indeed, a lot of genes implicated in this pathway have been identified in yeast and higher eukaryotes after the first discovery of the genetic regulation of the DNA damage checkpoint [12]. Even though it is easier to think about the DNA damage checkpoint as a linear pathway, as shown in Figure 1, where sensors act before transducers, which act before effectors and repair factors, the situation is far more complex as proteins that act at the very beginning of the cascade are also required for repairing and/or replicating the DNA. For instance, the Mre11-Rad50-Xrs2 (MRX-) complex in *S. cerevisiae* is one of the first recruited at the site of DSB and it is essential for starting both the checkpoint signal and the repair process [21,22]. Thus, there are likely extensive communications between the DNA damage checkpoint proteins and those involved in DNA replication and repair to ensure that not only the checkpoint is turned on in response to DNA damage but also it is properly turned off following DNA repair. 

### 2.1. Activation of the DNA Damage Checkpoint in Yeast

#### 2.1.1. Sensors: How the Signal Transduction Cascade Starts

**Figure 2:**
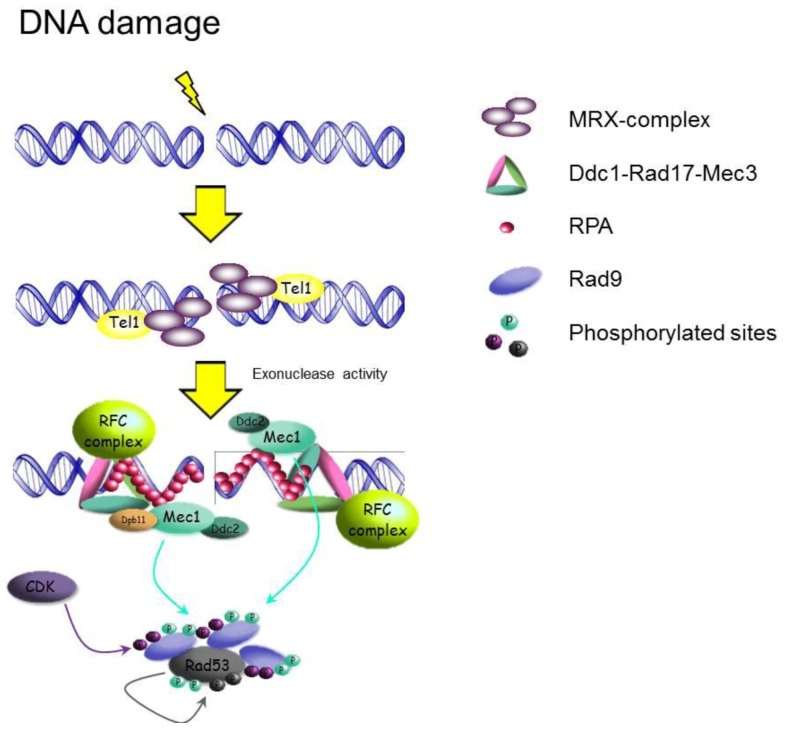
Schematic representation of the DNA damage checkpoint activation pathway in *S. cerevisiae* after DSBs in G2. For simplicity some factors are omitted.

As soon as DNA damage occurs, DNA damage checkpoint and repair proteins are able to form microscopically observable nuclear foci at the sites of damage. In particular, the MRX-complex is one of the first recruited at the site of DSBs. Its recruitment in yeast cells causes the co-localization of the protein kinase Tel1 at the site of damage [21]. Biochemical studies show that the MRN-complex (where Nbs1 is the mammalian ortholog of Xrs2) physically interacts with and stimulates the kinase activity of ATM (the mammalian ortholog of Tel1) in the presence of broken DNA ends [23,24]. Similarly, Tel1 can be activated by the interaction with MRX and DNA ends [25]. Both in yeast and mammals, DNA DSBs are quickly processed by nucleolytic resection into ssDNA which is coated by Replication Protein-A (RPA) [26,27,28]. RPA recruits the protein kinase Mec1 and its binding factor Ddc2 (ATR/ATRIP in mammal cells) (Figure 2), causing an ATM^Tel1^-to-ATR^Mec1^ switch that is cell cycle-dependent with occurrence restricted to the S and G2 phases [29]. Interestingly, in yeast Tel1 and MRX increase the efficiency of ssDNA generation, leading to Mec1-Ddc2 recruitment [22].

**Table 1:** DNA damage checkpoint related factors.

Function	*S. cerevisiae*	*H.* *sapiens*	*S.* *pombe*
*Sensors*			
9-1-1 complex	Ddc1	hRad9	Rad9
	Mec3	hHus1	Hus1
	Rad17	hRad1	Rad1
RFC-like clamp loader	Rad24	hRad17	Rad17
	Rfc2-5	hRfc2-5	Rfc2-5
MRX complex	Mre11	hMre11	Mre11
	Rad50	hRad50	Rad50
	Xrs2	hNbs1	Nbs1
BRCT-containing	Dpb11	TopBP1	Cut5
		BRCA1	
		hMdc1	
ss-DNA binding	RPA	RPA	RPA
*Transducers*			
PI3K-like kinases	Mec1-Ddc2	ATR-ATRIP	Rad3-Rad26
	Tel1	ATM	Tel1
*Adaptors*			
	Rad9	53BP1	Crb2
	Mrc1	CLSPN	Mrc1
*Effectors*			
Checkpoint kinases	Chk1	CHK1	Chk1
	Rad53	CHK2	Cds1

In order to properly activate Mec1, and subsequently the DDR, other protein complexes are required. For instance, the 9-1-1 complex, formed by Ddc1, Mec3 and Rad17 in yeast, is loaded onto ssDNA and dsDNA junctions by RFC-like clamp loaders [30]. A number of RFC-like clamp loaders have been identified to function in the DNA damage checkpoint, where the biggest subunit of the canonical replication RFC, Rfc1, is replaced by either Rad24, Elg1 or Ctf18 [31]. Rad24-RFC is specifically involved in loading the 9-1-1 complex and functions in the Rad9 pathway in DNA damage checkpoint [32,33,34] (Figure 2). The Ctf18-RFC complex has recently been implicated in the Mrc1 dependent DNA replication checkpoint [35], while the Elg1-RFC clamp loader plays a role in DNA replication and has a redundant role to Rad24-RFC and Ctf18-RFC in the DNA damage response [36,37,38,39]. 

Biochemical study has indicated that the *Xenopus* TopBP1, homolog of yeast Dpb11, could activate ATR *in vitro* [40]. It has been similarly shown in yeast that Dpb11 can also activate Mec1 *in vitro* [41,42]. However, mutation of the Mec1-activation domain in Dpb11 does not cause a significant defect in the activation of the protein-kinase Rad53 [43], a hallmark of DNA damage checkpoint activation in yeast. This suggests that alternative pathways exist for a proper activation of the DNA damage checkpoint. In fact, Dpb11 has been shown to bind to Ddc1 [44] through interaction mediated by the phosphorylation of Thr-602 on Ddc1, and that this interaction is important for full activation of Rad53 [45,46]. Ddc1 can also use its *C*-terminal tail to activate the kinase activity of Mec1 *in vitro* and it appears to act in parallel to Dpb11 in the G2/M phase [47].

#### 2.1.2. Adaptors and Activation of Downstream Effector kinases

Activated Mec1 and Tel1 directly phosphorylate the adaptor proteins Rad9 and Mrc1, which are both required for Rad53 activation *in vivo* in response to different kind of damages and in different phases of the cell cycle [48,49,50]. Both the adaptors are able to recruit the checkpoint kinases Rad53 and Dun1, via their FHA domains, to trigger their activation. In particular, Mrc1 interacts with Tof1 and Csm3, associates with DNA replication forks [51,52,53] and has been shown to promote Rad53 phosphorylation by Mec1 directly under DNA replication stress conditions [54]. In agreement with the biochemical findings, the co-localization of Mrc1 and Mec1 is sufficient to promote Rad53 activation [55]. On the other hand, Rad9 functions to control Rad53 activation throughout the entire cell cycle. Cell biological studies show that Rad9 and its orthologs Crb2 and 53BP1, from fission yeast and mammals respectively, localize to the sites of DNA damage by a direct interaction between the Tudor domain of Rad9/53BP1 and methylated histone [21,56,57]. However, mutation of the nucleosomal histone H3-Lys79 methylase *DOT1*, which is responsible for this particular histone modification in yeast, does not cause any appreciable checkpoint defects. Moreover, the BRCT domain of Rad9 is a phosphoprotein-binding domain that has been suggested to bind the phosphorylated Ser129 of H2A [58,59,60], but mutation to eliminate Ser129 phosphorylation of H2A does not cause a checkpoint defect, suggesting that additional ligands exist. Rad9 is also phosphorylated by CDK on multiple SP/TP sites, which has been suggested to be a mechanism of its function in S and G2/M phase. Mutation of 18 SP/TP sites in the *N*-terminal region of Rad9 causes a loss of its checkpoint activity [61] and recent studies have suggested that Dpb11 binds to Rad9 via its CDK phosphorylated sites [43] to recruit Rad9 to Mec1 and promote the adaptor phosphorylation by Mec1. However, latest results indicate that multiple additional CDK consensus sites in Rad9 are involved in its interaction with Dpb11. Surprisingly, the Dpb11-Rad9 interaction is even dispensable for checkpoint activation in the G2/M phase [62]. Therefore, the CDK regulation of checkpoint activation through Rad9 is likely more complex.

Upon recruitment to Rad9 and Mrc1, Mec1/Tel1 phosphorylates Rad53. Studies on Rad53, its ortholog Cds1 in fission yeast and Chk2 in mammals, have shown that phosphorylation of the *N*-terminal TQ sites of the Chk2 family kinase mediates its dimerization via the FHA domain [63], leading to its *trans*-phosphorylation on a conserved threonine residue in the activation loop and subsequent activation (Figure 2) [64]. Rad53 directly phosphorylates the activation loop of Dun1 for its activation [64] and the interaction between the FHA domain of Dun1 and *N*-terminal TQ phosphorylation sites of Rad53 is critical for this kinase-to-kinase trans-phosphorylation event [64,65,66].

### 2.2. Inactivation of the DNA Damage Checkpoint: How to Silence the Checkpoint

As activation of the DNA damage checkpoint is necessary for orchestrating cell cycle arrest, transcriptional regulation, and DNA repair, cells also need to properly inactivate the DNA damage checkpoint in order to resume cell cycle progression after repairing the lesions. The mechanism for the inactivation of the DNA damage checkpoint is known as ‘checkpoint recovery’. Presumably, inactivation of the checkpoint coincides with the removal of DNA lesions, although yeast cells have the ability to inactivate the checkpoint even in the presence of persistent DNA lesions through a phenomenon known as ‘checkpoint adaptation’ [67,68]. 

#### 2.2.1. Checkpoint Recovery: When It Is Safe to Switch the Checkpoint Off

Several genes that have been implicated in recovery defect are involved in distinct stages of the DNA damage checkpoint, suggesting that checkpoint recovery could be a multistep process rather than a single inactivation mechanism. Several mechanisms, not mutually exclusive, have been proposed including (1) disassembly of DNA repair and checkpoint proteins on the site of damage [69]; (2) removal of DNA lesions [70]; (3) recruitment of protein phosphatases [71,72], and (4) feedback control via transcriptional changes [73], as discussed further below.

Proper inactivation of the checkpoint requires the disassembly of the same mechanisms that are required for its activation. For instance, the removal of DNA lesions after repair would disengage the sensors and activators of the checkpoint, including Mec1, Tel1, the 9-1-1 and MRX complexes and would prevent them from further transducing the signal to Rad53. Genetic and biochemical analyses of Sae2 and Srs2, which are involved in DNA repair, have demonstrated that these factors are linked to checkpoint inactivation too. In particular, Sae2 is part of the initial sensing mechanism of DNA DSBs and works to promote DNA end resection with the MRX complex [74]. *SAE2* deletion or mutation of its Mec1/Tel1 phosphorylation sites have been shown to cause a more persistent checkpoint activation and cell cycle arrest that cannot be only correlated to repair defects [75,76]. Accordingly, over-expressed *SAE2* was also shown to antagonize checkpoint activation. This brings about the possibility that Mec1/Tel1-dependent phosphorylation of Sae2 is a way for mediating DNA repair with the DNA damage checkpoint, although the mechanism remains unknown. 

DNA structure also plays an important role in eliciting the DDR. There are several mechanisms the cell uses to identify DNA lesions and structures (e.g., 9-1-1 complex, MRX-complex, replication fork), which might be targeted during inactivation. For instance, during homologous recombination to repair DNA DSBs, the DNA resection machinery processes broken DNA ends to generate ssDNA 3’-tails, leading to Rad51 filament formation towards homology search and recombination. The presence of ssDNA is important to maintain the checkpoint before the repair has completed. Srs2, a DNA helicase with DNA translocation properties, acts as an anti-recombinase factor. In fact, loss of Srs2 not only abrogates checkpoint inactivation, but also results in persistent ssDNA and Rad51 association [69,70]. Nevertheless, the checkpoint recovery defect of *srs2∆* cells is more damage specific and so far only seen in the context of a DNA DSB using the HO-endonuclease induced system, whereas in *sae2∆* mutants checkpoint recovery defect has been shown in other types of damage. This suggests that different kinds of intermediate DNA structures during DNA repair could also trigger checkpoint activation.

Checkpoint recovery in yeast requires the inactivation of Rad53, which has to be degraded or dephosphorylated. Phosphatases Ptc2 and Ptc3 have been implicated in dephosphorylating Rad53 [77]. These phosphatases are part of the PP2C family and seem to share redundant roles in inactivating Rad53. In the HO-induced DSB system, over-expression of *PTC2* promotes Rad53 dephosphorylation and results in faster resumption of cell cycle progression [77]. Ptc2 interacts with Rad53 specifically through its FHA1 domain further supporting its role as the main phosphatase in Rad53 inactivation [77,78,79]. Pph3, another phosphatase, has also been implicated in Rad53 inactivation [71,72]. Since the mutations of all these phosphatases fail to completely impair Rad53 dephosphorylation, it might be that other phosphatases [80,81,82] or additional pathways are responsible for a complete checkpoint inactivation. 

Downstream targets of Rad53 could also provide a feedback mechanism to maintain checkpoint function and inactivation after DNA repair. Indeed, the lack of the downstream kinase Dun1 results in prolonged Rad53 activation after replication stress induced by hydroxyurea [73]. This Rad53 inactivation defect is further exacerbated in combination with *CCR4* deletion. Ccr4-Not complex is part of the transcriptional machinery and Ccr4 is the catalytic subunit of the mRNA deadenylase complex and regulates mRNA turnover. Ccr4 can affect transcriptional targets of Rad53 and Dun1, and has been demonstrated to influence the abundance of Crt1, a transcriptional repressor of DNA damage induced genes. Therefore, Ccr4 can also affect many other DNA damage induced transcriptional targets, which could provide a feedback control towards Rad53 and Dun1 inactivation.

#### 2.2.2. Checkpoint Adaptation: Escaping the Checkpoint in the Presence of Damage

Adaptation is the overriding of the checkpoint in presence of irreparable DNA lesions. Persistent DNA lesions, such as a DNA DSB or broken chromosome, allow continuous checkpoint activation as a consequence of the presence of active Mec1/Tel1 at the sites of damage [67,83,84]. Indeed, microscopy studies have shown that Ddc2-foci, a marker of Mec1-Ddc2 complex, correlates with active Rad53 [85]. Nevertheless, yeast cells eventually resume cell cycle progression even in the presence of DNA lesions suggesting that there exists an “active” process that is required to disengage Rad53 from Mec1 and Tel1 signaling and that allows the cells to adapt to the presence of dangerous lesions. Studies on adaptation have revealed numerous genes involved in this kind of checkpoint inactivation, such as *CDC5*, *CKB2*, *YKU70*, *SAE2* and *SRS2* [67,70,83,86]. Recent work has shown the polo-like kinase Cdc5 may play a pertinent role in facilitating checkpoint adaptation. In fact, over-expression of *CDC5* is able to suppress checkpoint activation in presence of an irreparable DSB and in *cdc13-1* mutants [83,84,86]. It remains to be seen whether Cdc5 kinase activity on potential targets such as Rad53 is part of the processing of adaptation. Moreover, adaptation also requires dissociation of the sensors of DNA damage suggesting that adaptation functions through multiple mechanisms of action. 

While a number of mechanisms have been proposed for checkpoint recovery and adaptation, ranging from the disassembly of activators for the checkpoint and recruitment of phosphatases, it is not known whether these mechanisms of action take place in a temporal manner or *en masse* once DNA lesions are repaired. Further studies are needed to reveal how the checkpoint activity is coupled to the status of DNA repair.

## 3. Genome Maintenance: Focusing on Telomere

Genetic studies have shown that Mec1 and Tel1 have a major role in the maintenance of genome integrity. For example, in yeast cells the single deletion of either *MEC1* or *TEL1* causes moderate to no increase in gross chromosomal rearrangements (GCRs); however, the inactivation of both kinases causes a synergistic increase in GCRs [87]. On the other hand, *tel1Δ* cells have shorter telomeres than wild type cells, while the deletion of *MEC1* has little effect on telomere length. Interestingly, the lack of both kinases causes telomere loss and chromosomal fusions [88,89]. One of the major unsolved questions is how Mec1 and Tel1 are involved in genome maintenance. In this context, a particularly relevant issue is the metabolism of telomeres, which must be recognized and maintained differently from deleterious DNA DSBs to prevent checkpoint activation and rearrangements at chromosome ends. 

### 3.1. Protection of Chromosome Ends: Telomeres

**Figure 3:**
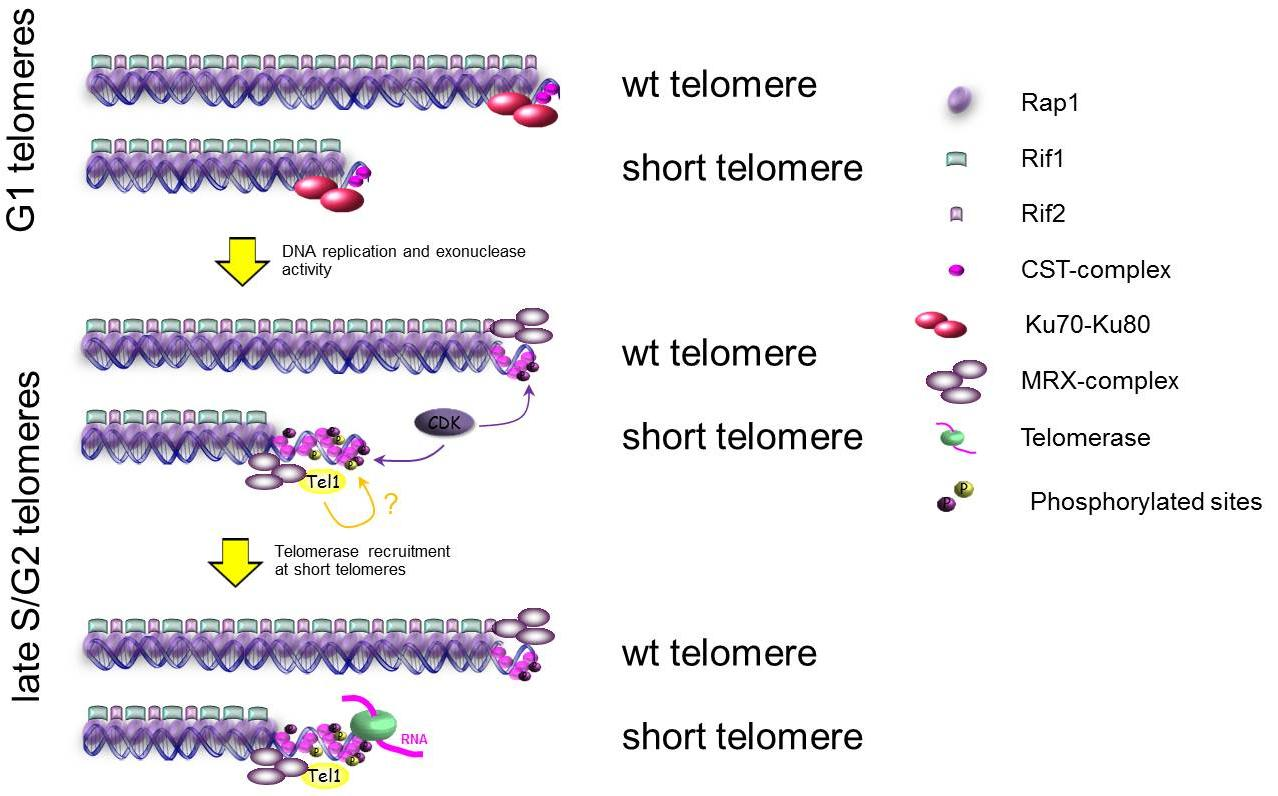
Schematic representation of the regulation of telomere lengthening in *S. cerevisiae*. For simplicity some factors are omitted.

Telomeres are nuclear-protein complexes at the end of eukaryotic chromosomes that protect from erosion by nucleases and ensure a correct and complete replication of the extremities of DNA [90]. Moreover, these structures distinguish natural chromosome ends from DNA DSBs, inhibiting the activation of the DNA damage checkpoint and repair processes at chromosome ends. In most eukaryotes, telomeric DNA consists of tandem repeats of a short sequence that extends from several hundred base-pairs (~300 bp in *Saccharomyces cerevisiae*) to thousands of base pairs in mammals. The 3’-strand is G-rich and is extended to form a single-stranded overhang known as the G-tail [90,91,92], which is bound *in vivo* by sequence specific DNA-binding proteins like Cdc13 in *S. cerevisiae* [93,94]. Additionally, Cdc13 is able to recruit other proteins like Stn1 and Ten1 [95,96,97,98], forming a heterotrimeric complex essential for protecting telomeres from nuclease activities and for recruiting the reverse transcriptase responsible of the G-strand synthesis, the telomerase [99]. The Cdc13-Stn1-Ten1 (CST) complex is specific for binding the ssDNA at the end of yeast telomeres and it is structurally similar to the RPA complex [97,98]. Telomeres are subject to continuous shortening due to the removal of the primers of the canonical semi-conservative DNA replication and nuclease erosion. In order to ensure the stability of chromosome ends, telomeres are replicated by telomerase, a specialized reverse transcriptase, which uses a specific RNA as a template to lengthen the telomeric G-tail (Figure 3). The complementary *C*-strand is synthesized via semi-conservative DNA replication. 

### 3.2. Replication of Telomeres: When and Where?

Telomere elongation is a well-controlled and dynamic process. It has been shown in *S. cerevisiae* that telomerase is active preferentially at shorter telomeres and only in late S/G2 phases of the cell cycle [100]. The major open questions are about how cells are able to recognize shorter telomeres for elongation and to lengthen these short telomeres only during late S/G2 phase? It has been demonstrated that before telomerase action, telomeres have to be resected to generate a G-tail that is the substrate for telomerase activity. The resection at telomeres requires the same nuclease machinery, responsible of DSBs processing, which includes Mre11, Exo1, Sgs1 and Dna2 [101,102,103]. A lot of data demonstrate that the resection of DSBs happens only in G2 and requires a high CDK activity [104], suggesting that one or more factors involved in the resection could be CDK substrates and regulated by CDK [105,106]. Experiments on *de novo* telomeres, generated after cutting an HO-site flanked by TG-repeats or after inducing recombination at telomeres, show that the resection happens only in late S/G2 phase [107]. In G1 this process is inhibited by Ku70/80-complex, which is also involved in inhibiting the resection at DSBs in G1 [76], and by a telomeric specific complex formed by Rap1-Rif1-Rif2 [108]. This complex, recently called Shelterin-like complex [90], is localized at telomeres and through Rap1 is bound directly to double strand telomeric DNA preserving this DNA from degradation [108,109] (Figure 3 Upper panel). At *de novo* telomeres, it looks like Rap1 and Rif2 are involved in inhibiting both the G- and the C-strand degradation in G1, while the role of Rif1 in this pathway is not so clear [107]. Moreover, it has been shown that the lack of Rif2, of the C-terminus of Rap1 or of Ku70 increases the ssDNA at native chromosome ends [107]. All these data suggest that the proteins involved in protecting chromosome ends from degradation, like Rap1, Rif2 or Ku70-Ku80, could be directly or indirectly the substrates of CDK to control the access of nucleases to telomeric DNA. Moreover, it has been shown that the recruitment of the telomerase components, Est1, Est2, Est3 and Cdc13, increases at telomeres in S/G2 phases [100,110,111], and this recruitment is strictly dependent on Est1, a cell cycle controlled recruiter and activator of telomerase [112]. Genetic and biochemical evidences strongly imply that telomerase recruitment to telomeres is achieved by specific interaction between Cdc13 and Est1 [113,114,115], and this interaction seems to be dependent on Cdc13 phosphorylation by CDK [114], which could contribute to a cell cycle regulated recruitment and activation of the telomerase.

Telomere lengthening is preferentially occurring at shorter telomeres in cells [116], but how do cells detect shorter telomeres? It has been proposed that Rap1, Rif1 and Rif2 are part of a “counting system” that measures telomere length by the number of these proteins present on each telomere (*in cis*) [108]. In fact, tethering Rap1, Rif1 or Rif2 at telomeres is able to inhibit telomere lengthening *in cis* [117]. However, at short telomeres Rif2, but not Rif1, occupancy is reduced [118], suggesting that Rif2 is more important than Rif1 in the “counting system”. Consistent with their inhibitory functions, *rif1Δ* mutants have longer telomeres than *rif2Δ* cells and the double mutants *rif1Δ rif2Δ* have telomeres that are longer than each single mutant, indicating that they are acting in two different pathways [119]. Since the recent involvement of Rif2 in protecting chromosome ends from nuclease degradation, it could be that a less amount of this protein at short telomeres allows nucleases to be recruited and activated in G2, as previously discussed, to generate the amount of ssDNA required for CST-complex binding and recruitment of telomerase, even though the molecular details of this mechanism are not yet elucidated. In this pathway, a crucial role is carried out by the protein kinase Tel1, that is recruited preferentially at short telomeres [120,121,122]. It has been shown that Tel1 and Rif2 are competing for the binding to Xrs2 C-terminal domain [118,123]. At short telomeres, where Rif2 is less abundant [120], Tel1 could be favored in binding Xrs2, leading to the recruitment of the kinase and, as a result, the phosphorylation of proteins that cause telomere lengthening (Figure 3). Different candidates have been proposed, for instance Cdc13, the specific telomeric ssDNA-binding protein, but the data about the function of its phosphorylation are controversial. In fact, even though it was proposed that Cdc13 phosphorylation by Mec1/Tel1 was essential for the interaction between Cdc13 and Est1 [124], the latest data indicate that the mutagenesis of every potential consensus phosphorylation site for Tel1 confers nearly wild-type telomeres length [115] and does not affect Cdc13-Est1 interaction [125]. Furthermore, it has been shown that Rif1 is phosphorylated in a Mec1/Tel1-dependent manner [126], but the function of this phosphorylation remains unknown.

### 3.3. The Ends of Chromosomes Are Not DSBs

Telomeres are naturally stable and are protected from DNA damage checkpoint, homologous recombination as well as end-to-end fusion that normally promote intrachromosomal DSBs repair [127]. It has been hypothesized that the TG-repeats and/or the protein complexes bound to the telomeric sequences exert an anti-checkpoint function at a DSB flanked by telomeric sequences inhibiting the recruitment and the activation of the checkpoint proteins at *de novo* telomeres [128,129]. Moreover, alterations at native telomeres caused by uncapping of telomeres, using *cdc13-1* mutant for instance, elicit DNA degradation that leads to accumulation of ssDNA. This ssDNA recruits RPA, Mec1/Ddc2 and triggers DNA damage checkpoint activation. Interestingly, deletion of Rif1 exacerbates the checkpoint activation of *cdc13-1* mutant, suggesting that Rif1 could inhibit the recruitment of DNA damage checkpoint proteins at damaged telomeres [130]. A similar function could be carried out by Cdc13-Stn1-Ten1 complex, which is able to bind specifically telomeric ssDNA [98], protecting it from RPA and Mec1 binding.

## 4. Conclusions

Extensive studies using yeast and other organisms have identified most of the genes involved in the DNA damage checkpoint and much of its genetic basis has been established. In some instances, certain steps of the DNA damage checkpoint activation have been reconstituted biochemically. However, many mechanistic questions remain unanswered upon closer examinations. For example, Rad9 is central to DNA damage checkpoint activation. Although Dpb11 interacts with CDK-phosphorylated Rad9 and was found to activate the Mec1 kinase, the abolishment of this interaction does not affect checkpoint activation *in vivo* [62]. Since CDK phosphorylation of Rad9 is essential for checkpoint activation, it is likely that there could be factors other than Dpn11 interacting with Rad9 for full checkpoint activation which remain to be identified.

Relatively little is known concerning the molecular basis of the inactivation of the DNA damage checkpoint following the completion of DNA repair. Although recent studies on checkpoint inactivation and a lot of evidences are suggesting that this is a genetically controlled pathway, it is not clear which are the crucial factors involved in turning off the checkpoint cascade after the repair of DNA damage. The difficulties in identifying these factors could be due to the redundancy of pathways and enzymes involved, like phosphatases or damage sensors themselves. Moreover, DNA damage sensors could have dual roles in activating the DNA damage checkpoint as well as serving as a feedback control to inactivate the checkpoint. One such example could be Sae2, a DNA repair protein that is known to be a negative regulator of the DNA damage checkpoint [76]. 

Recent data have elucidated some molecular mechanisms responsible for regulating telomere resection and elongation by cell cycle and telomere length by itself. Nevertheless, there are several steps of these pathways which need to be further investigated. For example, the role of the telomere lengthening inhibitor Rif1 has been genetically demonstrated. In fact, *rif1Δ* mutants have really long telomeres and cells overexpressing *RIF1* show short telomeres. However, it is completely unclear how this protein interferes with telomerase activity or cell cycle regulation of telomere lengthening and which factors Rif1 is interacting with to carry out this role.

Despite that numerous substrates have been found for Mec1 and Tel1 in yeast and ATR and ATM in mammalian cells by high throughput screenings [126,131,132], it is poorly understood how these kinases regulate telomere elongation and suppress chromosomal rearrangements. Considering that chromosomal rearrangements are a hallmark of many human diseases, especially cancers, understanding how they are prevented would provide new insights into the development of therapeutic strategies in the future. Finally, we apologize for those studies that are inadvertently omitted. 

## References

[1] Ciccia A., Elledge S.J. (2010). The DNA damage response: making it safe to play with knives. Mol. Cell.

[2] Zhou B.B., Elledge S.J. (2000). The DNA damage response: putting checkpoints in perspective. Nature.

[3] Shiloh Y. (2003). ATM and related protein kinases: safeguarding genome integrity. Nat. Rev. Cancer.

[4] Langerak P., Russell P. (2011). Regulatory networks integrating cell cycle control with DNA damage checkpoints and double-strand break repair. Philos. Trans. R. Soc. Lond. B Biol. Sci..

[5] Flynn R.L., Zou L. (2011). ATR: a master conductor of cellular responses to DNA replication stress. Trends Biochem. Sci..

[6] Lengauer C., Kinzler K.W., Vogelstein B. (1998). Genetic instabilities in human cancers. Nature.

[7] Kolodner R.D., Putnam C.D., Myung K. (2002). Maintenance of genome stability in *Saccharomyces cerevisiae*. Science.

[8] Vessey C.J., Norbury C.J., Hickson I.D. (1999). Genetic disorders associated with cancer predisposition and genomic instability. Prog. Nucleic Acid Res. Mol. Biol..

[9] Bayani J., Squire J.A. (2001). Advances in the detection of chromosomal aberrations using spectral karyotyping. Clin. Genet..

[10] Deininger P.L., Batzer M.A. (1999). Alu repeats and human disease. Mol. Genet. MeTable.

[11] Lindahl T. (1993). Instability and decay of the primary structure of DNA. Nature.

[12] Weinert T.A., Hartwell L.H. (1988). The *RAD9* gene controls the cell cycle response to DNA damage in *Saccharomyces cerevisiae*. Science.

[13] Hartwell L.H., Weinert T.A. (1989). Checkpoints: controls that ensure the order of cell cycle events. Science.

[14] Lowndes N.F., Murguia J.R. (2000). Sensing and responding to DNA damage. Curr. Opin. Genet. Dev..

[15] Bartek J., Lukas J. (2007). DNA damage checkpoints: from initiation to recovery or adaptation. Curr. Opin. Cell Biol..

[16] Longhese M.P., Foiani M., Muzi-Falconi M., Lucchini G., Plevani P. (1998). DNA damage checkpoint in budding yeast. EMBO J..

[17] Foiani M., Pellicioli A., Lopes M., Lucca C., Ferrari M., Liberi G., Muzi-Falconi M., Plevani1 P. (2000). DNA damage checkpoints and DNA replication controls in *Saccharomyces cerevisiae*. Mutat. Res..

[18] Finn K., Lowndes N.F., Grenon M. (2012). Eukaryotic DNA damage checkpoint activation in response to double-strand breaks. Cell Mol. Life Sci..

[19] Harper J.W., Elledge S.J. (2007). The DNA damage response: ten years after. Mol. Cell.

[20] Vinella D., D'Ari R. (1995). Overview of controls in the *Escherichia coli* cell cycle. Bioessays..

[21] Lisby M., Barlow J.H., Burgess R.C., Rothstein R. (2004). Choreography of the DNA damage response: spatiotemporal relationships among checkpoint and repair proteins. Cell.

[22] Mantiero D., Clerici M., Lucchini G., Longhese M.P. (2007). Dual role for *Saccharomyces cerevisiae* Tel1 in the checkpoint response to double-strand breaks. EMBO Rep..

[23] Lee J.-H., Paull T.T. (2004). Direct activation of the ATM protein kinase by the Mre11/Rad50/Nbs1 complex. Science.

[24] Lee J.-H., Paull T.T. (2005). ATM activation by DNA double-strand breaks through the Mre11-Rad50-Nbs1 complex. Science.

[25] Fukunaga K., Kwon Y., Sung P., Sugimoto K. (2011). Activation of protein kinase Tel1 through recognition of protein-bound DNA ends. Mol. Cell Biol..

[26] Shiotani B., Zou L. (2009). Single-stranded DNA orchestrates an ATM-to-ATR switch at DNA breaks. Mol. Cell.

[27] You Z., Chahwan C., Bailis J., Hunter T., Russell P. (2005). ATM activation and its recruitment to damaged DNA require binding to the C terminus of Nbs1. Mol. Cell Biol..

[28] Zou L., Elledge S.J. (2003). Sensing DNA damage through ATRIP recognition of RPA-ssDNA complexes. Science.

[29] Jazayeri A., Falck J., Lukas C., Bartek J., Smith G.C. M., Lukas J., Jackson S.P. (2006). ATM- and cell cycle-dependent regulation of ATR in response to DNA double-strand breaks. Nat. Cell Biol..

[30] Kondo T., Matsumoto K., Sugimoto K. (1999). Role of a complex containing Rad17, Mec3, and Ddc1 in the yeast DNA damage checkpoint pathway. Mol. Cell Biol..

[31] Majka J., Burgers P.M. J. (2004). The PCNA-RFC families of DNA clamps and clamp loaders. Prog. Nucleic Acid Res. Mol. Biol..

[32] Lydall D., Weinert T. (1997). G2/M checkpoint genes of *Saccharomyces cerevisiae*: Further evidence for roles in DNA replication and/or repair. Mol. Gen. Genet..

[33] de M.A., Green C.M., Lowndes N.F. (1998). *RAD9* and *RAD24* define two additive, interacting branches of the DNA damage checkpoint pathway in budding yeast normally required for Rad53 modification and activation. EMBO J..

[34] Green C.M., Erdjument-Bromage H., Tempst P., Lowndes N.F. (2000). A novel Rad24 checkpoint protein complex closely related to replication factor C. Curr. Biol..

[35] Crabbé L., Thomas A., Pantesco V., De Vos J., Pasero P., Lengronne A. (2010). Analysis of replication profiles reveals key role of RFC-Ctf18 in yeast replication stress response. Nat. Struct. Mol. Biol..

[36] Bellaoui M., Chang M., Ou J., Xu H., Boone C., Brown G.W. (2003). Elg1 forms an alternative RFC complex important for DNA replication and genome integrity. EMBO J..

[37] Ben-Aroya S., Koren A., Liefshitz B., Steinlauf R., Kupiec M. (2003). *ELG1*, a yeast gene required for genome stability, forms a complex related to replication factor C. Proc. Natl. Acad. Sci. USA.

[38] Aroya S.B., Kupiec M. (2005). The Elg1 replication factor C-like complex: A novel guardian of genome stability. DNA Repair (Amst.).

[39] Kanellis P., Agyei R., Durocher D. (2003). Elg1 forms an alternative PCNA-interacting RFC complex required to maintain genome stability. Curr. Biol..

[40] Kumagai A., Lee J., Yoo H.Y., Dunphy W.G. (2006). TopBP1 activates the ATR-ATRIP complex. Cell.

[41] Mordes D.A., Nam E.A., Cortez D. (2008). Dpb11 activates the Mec1-Ddc2 complex. Proc. Natl. Acad. Sci. USA.

[42] Navadgi-Patil V.M., Burgers P.M. (2008). Yeast DNA replication protein Dpb11 activates the Mec1/ATR checkpoint kinase. J. Biol. Chem..

[43] Pfander B., Diffley J.F. X. Dpb11 coordinates Mec1 kinase activation with cell cycle-regulated Rad9 recruitment. EMBO J..

[44] Wang H., Elledge S.J. (2002). Genetic and physical interactions between *DPB11* and *DDC*1 in the yeast DNA damage response pathway. Genetics.

[45] Puddu F., Granata M., Di Nola L., Balestrini A., Piergiovanni G., Lazzaro F., Giannattasio M., Plevani P., Muzi-Falconi M. (2008). Phosphorylation of the budding yeast 9-1-1 complex is required for Dpb11 function in the full activation of the UV-induced DNA damage checkpoint. Mol. Cell Biol..

[46] Longhese M.P., Paciotti V., Fraschini R., Zaccarini R., Plevani P., Lucchini G. (1997). The novel DNA damage checkpoint protein Ddc1p is phosphorylated periodically during the cell cycle and in response to DNA damage in budding yeast. EMBO J..

[47] Navadgi-Patil V.M., Burgers P.M. (2009). The unstructured C-terminal tail of the 9-1-1 clamp subunit Ddc1 activates Mec1/ATR via two distinct mechanisms. Mol. Cell.

[48] Tanaka K., Russell P. (2001). Mrc1 channels the DNA replication arrest signal to checkpoint kinase Cds1. Nat. Cell Biol..

[49] Alcasabas A.A., Osborn A.J., Bachant J., Hu F., Werler P.J., Bousset K., Furuya K., Diffley J.F., Carr A.M., Elledge S.J. (2001). Mrc1 transduces signals of DNA replication stress to activate Rad53. Nat. Cell Biol..

[50] Schwartz M.F., Duong J.K., Sun Z., Morrow J.S., Pradhan D., Stern D.F. (2002). Rad9 phosphorylation sites couple Rad53 to the *Saccharomyces cerevisiae* DNA damage checkpoint. Mol. Cell.

[51] Bando M., Katou Y., Komata M., Tanaka H., Itoh T., Sutani T., Shirahige K. (2009). Csm3, Tof1, and Mrc1 form a heterotrimeric mediator complex that associates with DNA replication forks. J. Biol. Chem..

[52] Tourrière H., Versini G., Cordón-Preciado V., Alabert C., Pasero P. (2005). Mrc1 and Tof1 promote replication fork progression and recovery independently of Rad53. Mol. Cell.

[53] Szyjka S.J., Viggiani C.J., Aparicio O.M. (2005). Mrc1 is required for normal progression of replication forks throughout chromatin in *S. cerevisiae*. Mol. Cell.

[54] Chen S.-H., Zhou H. (2009). Reconstitution of Rad53 activation by Mec1 through adaptor protein Mrc1. J. Biol. Chem..

[55] Berens T.J., Toczyski D.P. (2012). Colocalization of Mec1 and Mrc1 is sufficient for Rad53 phosphorylation *in vivo*. Mol. Biol. Cell.

[56] Du L.-L., Nakamura T.M., Russell P. (2006). Histone modification-dependent and -independent pathways for recruitment of checkpoint protein Crb2 to double-strand breaks. Genes Dev..

[57] Huyen Y., Zgheib O., Ditullio R.A., Gorgoulis V.G., Zacharatos P., Petty T.J., Sheston E.A., Mellert H.S., Stavridi E.S., Halazonetis T.D. (2004). Methylated lysine 79 of histone H3 targets 53BP1 to DNA double-strand breaks. Nature.

[58] Manke I.A., Lowery D.M., Nguyen A., Yaffe M.B. (2003). BRCT repeats as phosphopeptide-binding modules involved in protein targeting. Science.

[59] Yu X., Chini C.C. S., He M., Mer G., Chen J. (2003). The BRCT domain is a phospho-protein binding domain. Science.

[60] Hammet A., Magill C., Heierhorst J., Jackson S.P. (2007). Rad9 BRCT domain interaction with phosphorylated H2AX regulates the G1 checkpoint in budding yeast. EMBO Rep..

[61] Bonilla C.Y., Melo J.A., Toczyski D.P. (2008). Colocalization of sensors is sufficient to activate the DNA damage checkpoint in the absence of damage. Mol. Cell.

[62] Wang G., Tong X., Weng S., Zhou H. (2012). Multiple phosphorylation of Rad9 by CDK is required for DNA damage checkpoint activation. Cell Cycle.

[63] Lee S.J., Schwartz M.F., Duong J.K., Stern D.F. (2003). Rad53 phosphorylation site clusters are important for Rad53 regulation and signaling. Mol. Cell Biol..

[64] Chen S.-H., Smolka M.B., Zhou H. (2007). Mechanism of Dun1 activation by Rad53 phosphorylation in *Saccharomyces cerevisiae*. J. Biol. Chem..

[65] Bashkirov V.I., Bashkirova E.V., Haghnazari E., Heyer W.D. (2003). Direct kinase-to-kinase signaling mediated by the FHA phosphoprotein recognition domain of the Dun1 DNA damage checkpoint kinase. Mol. Cell Biol..

[66] Lee H., Yuan C., Hammet A., Mahajan A., Chen E.S.-W., Wu M.-R., Su M.-I., Heierhorst J., Tsai M.-D. (2008). Diphosphothreonine-specific interaction between an SQ/TQ cluster and an FHA domain in the Rad53-Dun1 kinase cascade. Mol. Cell.

[67] Lee S.E., Pellicioli A., Demeter J., Vaze M.P., Gasch A.P., Malkova A., Brown P.O., Botstein D., Stearns T., Foiani M., Haber J.E. (2000). Arrest, adaptation, and recovery following a chromosome double-strand break in *Saccharomyces cerevisia*. Cold Spring Harb. Symp. Quant. Biol..

[68] Clémenson C., Marsolier-Kergoat M.C. (2009). DNA damage checkpoint inactivation: adaptation and recovery. DNA Repair.

[69] Vaze M.B., Pellicioli A., Lee S.E., Ira G., Liberi G., Arbel-Eden A., Foiani M., Haber J.E. (2002). Recovery from checkpoint-mediated arrest after repair of a double-strand break requires Srs2 helicase. Mol. Cell.

[70] Yeung M., Durocher D. (2011). Srs2 enables checkpoint recovery by promoting disassembly of DNA damage foci from chromatin. DNA Repair.

[71] Keogh M.-C., Kim J.-A., Downey M., Fillingham J., Chowdhury D., Harrison J.C., Onishi M., Datta N., Galicia S., Emili A., Lieberman J., Shen X., Buratowski S., Haber J.E., Durocher D., Greenblatt J.F., Krogan N.J. (2006). A phosphatase complex that dephosphorylates gammaH2AX regulates DNA damage checkpoint recovery. Nature.

[72] O'Neill B.M., Szyjka S.J., Lis E.T., Bailey A.O., Yates J.R., Aparicio O.M., Romesberg F.E. (2007). Pph3-Psy2 is a phosphatase complex required for Rad53 dephosphorylation and replication fork restart during recovery from DNA damage. Proc. Natl. Acad. Sci. U.S.A..

[73] Woolstencroft R.N., Beilharz T.H., Cook M.A., Preiss T., Durocher D., Tyers M. (2006). Ccr4 contributes to tolerance of replication stress through control of *CRT1* mRNA poly(A) tail length. J. Cell Sci..

[74] Clerici M., Mantiero D., Lucchini G., Longhese M.P. (2005). The *Saccharomyces cerevisiae* Sae2 protein promotes resection and bridging of double strand break ends. J. Biol. Chem..

[75] Baroni E., Viscardi V., Cartagena-Lirola H., Lucchini G., Longhese M.P. (2004). The functions of budding yeast Sae2 in the DNA damage response require Mec1- and Tel1-dependent phosphorylation. Mol. Cell Biol..

[76] Clerici M., Mantiero D., Lucchini G., Longhese M.P. (2006). The *Saccharomyces cerevisiae* Sae2 protein negatively regulates DNA damage checkpoint signalling. EMBO Rep..

[77] Leroy C., Lee S.E., Vaze M.B., Ochsenbein F., Ochsenbien F., Guérois R., Haber J.E., Marsolier-Kergoat M.-C. (2003). PP2C phosphatases Ptc2 and Ptc3 are required for DNA checkpoint inactivation after a double-strand break. Mol. Cell.

[78] Smolka M.B., Chen S.H., Maddox P.S., Enserink J.M., Albuquerque C.P., Wei X.X., Desai A., Kolodner R.D., Zhou H. (2006). An FHA domain-mediated protein interaction network of Rad53 reveals its role in polarized cell growth. J. Cell Biol..

[79] Guillemain G., Ma E., Mauger S., Miron S., Thai R., Guérois R., Ochsenbein F., Marsolier-Kergoat M.-C. (2007). Mechanisms of checkpoint kinase Rad53 inactivation after a double-strand break in *Saccharomyces cerevisiae*. Mol. Cell Biol..

[80] Bazzi M., Mantiero D., Trovesi C., Lucchini G., Longhese M.P. (2010). Dephosphorylation of gamma H2A by Glc7/protein phosphatase 1 promotes recovery from inhibition of DNA replication. Mol. Cell Biol..

[81] Travesa A., Duch A., Quintana D.G. (2008). Distinct phosphatases mediate the deactivation of the DNA damage checkpoint kinase Rad53. J. Biol. Chem..

[82] Kim J.-A., Hicks W.M., Li J., Tay S.Y., Haber J.E. (2011). Protein phosphatases Pph3, Ptc2, and Ptc3 play redundant roles in DNA double-strand break repair by homologous recombination. Mol. Cell Biol..

[83] Toczyski D.P., Galgoczy D.J., Hartwell L.H. (1997). *CDC5* and CKII control adaptation to the yeast DNA damage checkpoint. Cell.

[84] Vidanes G.M., Sweeney F.D., Galicia S., Cheung S., Doyle J.P., Durocher D., Toczyski D.P. (2010). *CDC5* inhibits the hyperphosphorylation of the checkpoint kinase Rad53, leading to checkpoint adaptation. PLoS Biol..

[85] Tercero J.A., Longhese M.P., Diffley J.F. X. (2003). A central role for DNA replication forks in checkpoint activation and response. Mol. Cell.

[86] Pellicioli A., Lee S.E., Lucca C., Foiani M., Haber J.E. (2001). Regulation of *Saccharomyces* Rad53 checkpoint kinase during adaptation from DNA damage-induced G2/M arrest. Mol. Cell.

[87] Myung K., Datta A., Kolodner R.D. (2001). Suppression of spontaneous chromosomal rearrangements by S phase checkpoint functions in *Saccharomyces cerevisiae*. Cell.

[88] Craven R.J., Greenwell P.W., Dominska M., Petes T.D. (2002). Regulation of genome stability by *TEL1* and *MEC1*, yeast homologs of the mammalian ATM and ATR genes. Genetics.

[89] Mieczkowski P.A., Mieczkowska J.O., Dominska M., Petes T.D. (2003). Genetic regulation of telomere-telomere fusions in the yeast *Saccharomyces cerevisae*. Proc. Natl. Acad. Sci. USA.

[90] Smogorzewska A., de Lange T. (2004). Regulation of telomerase by telomeric proteins. Annu. Rev. Biochem..

[91] Vega L.R., Mateyak M.K., Zakian V.A. (2003). Getting to the end: telomerase access in yeast and humans. Nat. Rev. Mol. Cell Biol..

[92] Verdun R.E., Karlseder J. (2007). Replication and protection of telomeres. Nature.

[93] Nugent C.I., Hughes T.R., Lue N.F., Lundblad V. (1996). Cdc13p: a single-strand telomeric DNA-binding protein with a dual role in yeast telomere maintenance. Science.

[94] Lin J.J., Zakian V.A. (1996). The *Saccharomyces CDC13* protein is a single-strand TG1-3 telomeric DNA-binding protein* in vitro* that affects telomere behavior* in vivo*. Proc. Natl. Acad. Sci. USA.

[95] Grandin N., Damon C., Charbonneau M. (2001). Ten1 functions in telomere end protection and length regulation in association with Stn1 and Cdc13. EMBO J..

[96] Grandin N., Reed S.I., Charbonneau M. (1997). Stn1, a new *Saccharomyces cerevisiae* protein, is implicated in telomere size regulation in association with Cdc13. Genes Dev..

[97] Sun J., Yu E.Y., Yang Y., Confer L.A., Sun S.H., Wan K., Lue N.F., Lei M. (2009). Stn1-Ten1 is an Rpa2-Rpa3-like complex at telomeres. Genes Dev..

[98] Gao H., Cervantes R.B., Mandell E.K., Otero J.H., Lundblad V. (2007). RPA-like proteins mediate yeast telomere function. Nat. Struct. Mol. Biol..

[99] Pennock E., Buckley K., Lundblad V. (2001). Cdc13 delivers separate complexes to the telomere for end protection and replication. Cell.

[100] Taggart A.K. P., Teng S.-C., Zakian V.A. (2002). Est1p as a cell cycle-regulated activator of telomere-bound telomerase. Science.

[101] Bonetti D., Martina M., Clerici M., Lucchini G., Longhese M.P. (2009). Multiple pathways regulate 3' overhang generation at *S. cerevisiae* telomeres. Mol. Cell.

[102] Zhu Z., Chung W.-H., Shim E.Y., Lee S.E., Ira G. (2008). Sgs1 helicase and two nucleases Dna2 and Exo1 resect DNA double-strand break ends. Cell.

[103] Mimitou E.P., Symington L.S. (2008). Sae2, Exo1 and Sgs1 collaborate in DNA double-strand break processing. Nature.

[104] Ira G., Pellicioli A., Balijja A., Wang X., Fiorani S., Carotenuto W., Liberi G., Bressan D., Wan L., Hollingsworth N.M., Haber J.E., Foiani M. (2004). DNA end resection, homologous recombination and DNA damage checkpoint activation require CDK1. Nature.

[105] Chen X., Niu H., Chung W.-H., Zhu Z., Papusha A., Shim E.Y., Lee S.E., Sung P., Ira G. (2011). Cell cycle regulation of DNA double-strand break end resection by Cdk1-dependent Dna2 phosphorylation. Nat. Struct. Mol. Biol..

[106] Huertas P., Cortés-Ledesma F., Sartori A.A., Aguilera A., Jackson S.P. (2008). CDK targets Sae2 to control DNA-end resection and homologous recombination. Nature.

[107] Bonetti D., Clerici M., Anbalagan S., Martina M., Lucchini G., Longhese M.P. (2010). Shelterin-like proteins and Yku inhibit nucleolytic processing of *Saccharomyces cerevisiae* telomeres. PLoS Genet..

[108] Marcand S., Wotton D., Gilson E., Shore D. (1997). Rap1p and telomere length regulation in yeast. Ciba Found. Symp..

[109] Vodenicharov M.D., Laterreur N., Wellinger R.J. (2010). Telomere capping in non-dividing yeast cells requires Yku and Rap1. EMBO J..

[110] Fisher T.S., Taggart A.K. P., Zakian V.A. (2004). Cell cycle-dependent regulation of yeast telomerase by Ku. Nat. Struct. Mol. Biol..

[111] Tuzon C.T., Wu Y., Chan A., Zakian V.A. (2011). The *Saccharomyces cerevisiae* telomerase subunit Est3 binds telomeres in a cell cycle- and Est1-dependent manner and interacts directly with Est1* in vitro*. PLoS Genet..

[112] Chan A., Boulé J.-B., Zakian V.A. (2008). Two pathways recruit telomerase to *Saccharomyces cerevisiae* telomeres. PLoS Genet..

[113] Qi H., Zakian V.A. (2000). The *Saccharomyces* telomere-binding protein Cdc13p interacts with both the catalytic subunit of DNA polymerase alpha and the telomerase-associated est1 protein. Genes Dev..

[114] Li S., Makovets S., Matsuguchi T., Blethrow J.D., Shokat K.M., Blackburn E.H. (2009). Cdk1-dependent phosphorylation of Cdc13 coordinates telomere elongation during cell-cycle progression. Cell.

[115] Gao H., Toro T.B., Paschini M., Braunstein-Ballew B., Cervantes R.B., Lundblad V. (2010). Telomerase recruitment in *Saccharomyces cerevisiae* is not dependent on Tel1-mediated phosphorylation of Cdc13. Genetics.

[116] Teixeira M.T., Arneric M., Sperisen P., Lingner J. (2004). Telomere length homeostasis is achieved via a switch between telomerase- extendible and -nonextendible states. Cell.

[117] Levy D.L., Blackburn E.H. (2004). Counting of Rif1p and Rif2p on *Saccharomyces cerevisiae* telomeres regulates telomere length. Mol. Cell Biol..

[118] McGee J.S., Phillips J.A., Chan A., Sabourin M., Paeschke K., Zakian V.A. (2010). Reduced Rif2 and lack of Mec1 target short telomeres for elongation rather than double-strand break repair. Nat. Struct. Mol. Biol..

[119] Wotton D., Shore D. (1997). A novel Rap1p-interacting factor, Rif2p, cooperates with Rif1p to regulate telomere length in *Saccharomyces cerevisia*. Genes Dev..

[120] Sabourin M., Tuzon C.T., Zakian V.A. (2007). Telomerase and Tel1p preferentially associate with short telomeres in *S. cerevisiae*. Mol. Cell.

[121] Hector R.E., Shtofman R.L., Ray A., Chen B.-R., Nyun T., Berkner K.L., Runge K.W. (2007). Tel1p preferentially associates with short telomeres to stimulate their elongation. Mol. Cell.

[122] Bianchi A., Shore D. (2007). Increased association of telomerase with short telomeres in yeast. Genes Dev..

[123] Hirano Y., Fukunaga K., Sugimoto K. (2009). Rif1 and Rif2 inhibit localization of tel1 to DNA ends. Mol. Cell.

[124] Tseng S.-F., Lin J.-J., Teng S.-C. (2006). The telomerase-recruitment domain of the telomere binding protein Cdc13 is regulated by Mec1p/Tel1p-dependent phosphorylation. Nucleic Acids Res..

[125] Wu Y., Zakian V.A. (2011). The telomeric Cdc13 protein interacts directly with the telomerase subunit Est1 to bring it to telomeric DNA ends *in vitro*. Proc. Natl. Acad. Sci. USA.

[126] Smolka M.B., Albuquerque C.P., Chen S.H., Zhou H. (2007). Proteome-wide identification of *in vivo* targets of DNA damage checkpoint kinases. Proc. Natl. Acad. Sci. USA..

[127] Longhese M.P. (2008). DNA damage response at functional and dysfunctional telomeres. Genes Dev..

[128] Ribeyre C., Shore D. (2012). Anticheckpoint pathways at telomeres in yeast. Nat. Struct. Mol. Biol..

[129] Michelson R.J., Rosenstein S., Weinert T. (2005). A telomeric repeat sequence adjacent to a DNA double-stranded break produces an anticheckpoint. Genes Dev..

[130] Xue Y., Rushton M.D., Maringele L. (2011). A novel checkpoint and RPA inhibitory pathway regulated by Rif1. PLoS Genet..

[131] Chen S.H., Albuquerque C.P., Liang J., Suhandynata R.T., Zhou H. (2010). A proteome-wide analysis of kinase-substrate network in the DNA damage response. J. Biol. Chem..

[132] Matsuoka S., Ballif B.A., Smogorzewska A., McDonald E.R., Hurov K.E., Luo J., Bakalarski C.E., Zhao Z., Solimini N., Lerenthal Y., Shiloh Y., Gygi S.P., Elledge S.J. (2007). ATM and ATR substrate analysis reveals extensive protein networks responsive to DNA damage. Science.

